# Critical Roles of Calpastatin in Ischemia/Reperfusion Injury in Aged Livers

**DOI:** 10.3390/cells10081863

**Published:** 2021-07-23

**Authors:** Joseph Flores-Toro, Sung-Kook Chun, Jun-Kyu Shin, Joan Campbell, Melissa Lichtenberger, William Chapman, Ivan Zendejas, Kevin Behrns, Christiaan Leeuwenburgh, Jae-Sung Kim

**Affiliations:** 1 Department of Surgery, University of Florida, Gainesville, FL 32610, USA; josephflorestoro@yahoo.com (J.F.-T.); sungkoc@uci.edu (S.-K.C.); ivanzendejas76@gmail.com (I.Z.); kevin.behrns@surgery.ufl.edu (K.B.); 2 Department of Surgery, Washington University in St. Louis, St. Louis, MO 63110, USA; junkyu@wustl.edu (J.-K.S.); jcampbell25@wustl.edu (J.C.); lichtenberger@wustl.edu (M.L.); chapmanw@wustl.edu (W.C.); 3 Department of Aging and Geriatric Research, University of Florida, Gainesville, FL 32610, USA; cleeuwen@ufl.edu; 4 Department of Cell Biology and Physiology, Washington University in St. Louis, St. Louis, MO 63110, USA

**Keywords:** liver, ischemia/reperfusion, autophagy, mitochondria, calpastatin

## Abstract

Ischemia/reperfusion (I/R) injury unavoidably occurs during hepatic resection and transplantation. Aged livers poorly tolerate I/R during surgical treatment. Although livers have a powerful endogenous inhibitor of calpains, calpastatin (CAST), I/R activates calpains, leading to impaired autophagy, mitochondrial dysfunction, and hepatocyte death. It is unknown how I/R in aged livers affects CAST. Human and mouse liver biopsies at different ages were collected during in vivo I/R. Hepatocytes were isolated from 3-month- (young) and 26-month-old (aged) mice, and challenged with short in vitro simulated I/R. Cell death, protein expression, autophagy, and mitochondrial permeability transition (MPT) between the two age groups were compared. Adenoviral vector was used to overexpress CAST. Significant cell death was observed only in reperfused aged hepatocytes. Before the commencement of ischemia, CAST expression in aged human and mouse livers and mouse hepatocytes was markedly greater than that in young counterparts. However, reperfusion substantially decreased CAST in aged human and mouse livers. In hepatocytes, reperfusion rapidly depleted aged cells of CAST, cleaved autophagy-related protein 5 (ATG5), and induced defective autophagy and MPT onset, all of which were blocked by CAST overexpression. Furthermore, mitochondrial morphology was shifted toward an elongated shape with CAST overexpression. In conclusion, CAST in aged livers is intrinsically short-lived and lost after short I/R. CAST depletion contributes to age-dependent liver injury after I/R.

## 1. Introduction

Life expectancy has markedly increased over the past century and is projected to continue to increase. Extended life span is accompanied by more aging-mediated comorbidities, resulting in the steady growth of elderly patients who need surgery. More than half of the population over the age of 65 are estimated to receive surgical treatments once in their lives [1]. Generally, deficits in hepatic morphology and function with advancing age are less apparent clinically than in other organs. However, livers from elderly patients have poorer recovery from surgical stress during liver resection and transplantation, signifying reduced reparative capacity with aging [2,3,4,5]. The liver is innately vulnerable to hypoxic and anoxic stress, especially in pericentral regions. A temporary cessation and subsequent resumption of hepatic blood supply during liver surgery inevitably induce ischemia/reperfusion (I/R) injury. Mechanisms behind hepatic I/R injury are multifactorial, including overproduction of reactive oxygen species (ROS) and nitrogen species, Ca^2+^ deregulation, loss of cellular antioxidants, stimulation of catabolic enzymes, impaired autophagy, and mitochondrial dysfunction, all of which happen during the early phase of reperfusion [6]. Moreover, these early incidents incite hepatic inflammation during the late phase of reperfusion.

The onset of mitochondrial permeability transition (MPT) is a hallmark of mitochondrial dysfunction that develops soon after reperfusion [7]. Once high-conductance permeability transition pores open in mitochondrial inner membranes, mitochondrial oxidative phosphorylation uncouples and bioenergetic failure is followed [7,8,9,10]. We have demonstrated that timely clearance of dysfunctional mitochondria through autophagy, a cellular process eliminating abnormal cellular constituents and organelles [4], is indispensable for hepatocyte survival after I/R [2,11]. Besides mitochondrial autophagy (mitophagy), mitochondrial quality is also controlled by adaptive morphological changes in mitochondria, a phenomenon termed mitochondrial dynamics (MD) [12]. Under non-lethal stresses, healthy mitochondrion rapidly fuses with abnormal mitochondrion to compensate for defects of the latter, implicating that mitochondrial interconnection serves as the first line of defense against acute stress [13]. When abnormal mitochondria escape this fusion process, mitophagy removes them later [14]. Hence, MD and mitophagy synergistically control the mitochondrial quality.

Therapeutic strategies aiming at the downstream events to the MPT likely provide only temporary relief as the opening of mitochondrial pores still prevails upon reperfusion. Significant and persistent levels of cytoprotection against I/R injury would be achieved by blocking cellular abnormalities upstream to the MPT. One event provoking MPT onset after I/R is Ca^2+^ overload [11,15,16]. Importantly, the unrestricted elevation of Ca^2+^ stimulates calpains. The uncontrolled activation of calpains causes irreversible tissue injury by hydrolyzing substrates essential to cell survival, including autophagy proteins [17,18]. Nonetheless, under non-disease conditions, over-stimulation of calpains rarely occurs due to the presence of calpastatin (CAST), an endogenous calpain-specific inhibitor [19]. Questions, hence, arise as to how calpains could be stimulated after I/R, even though the liver has such a powerful endogenous inhibitor of calpains. It is currently unknown how I/R affects CAST in aged livers.

Here, using human and mouse livers and hepatocytes, we show that CAST in aged livers is intrinsically short-lived and is rapidly depleted after I/R, thereby leading to the stimulation of calpains, cleavage of autophagy-related protein 5 (ATG5), impaired autophagy, MPT onset, and ultimately hepatocyte death. Additionally, we uncover a new role of CAST in the regulation of mitochondrial shape.

## 2. Materials and Methods

### 2.1. Reagents

Tetramethylrhodamine methylester (TMRM), calcein/AM, rhodamine-123 (Rd-123), and propidium iodide (PI) were purchased from ThermoFisher Scientific (Pittsburgh, PA, USA). Embedding agents for transmission electron microscopy were purchased from Electron Microscopy Sciences (Hatfield, PA, USA). All other chemicals were purchased from Millipore Sigma (St. Louise, MO, USA) except if noted otherwise.

### 2.2. Human Liver Biopsy Collection

Human liver biopsies were collected from the patients undergoing hepatic resection surgery in accordance with the protocol reviewed and approved by the Institutional Review Board of the University of Florida (201400182) and the Washington University in St. Louis (201902153). Twelve adult patients diagnosed with hepatocellular cancer were included in the present study (Supplemental Table S1).

### 2.3. Hepatocyte Isolation and Culture

To compare the similarities and differences in response to I/R between human and rodent livers, mouse livers and hepatocytes were subjected to I/R. Animals received humane care according to the protocols approved by the Institutional Care and Use Committee of the University of Florida (201202396) and Washington University in St. Louis (20190005). Three-month-old male C57BL/6 J mice were purchased from the Jackson Laboratory (Bar Harbor, ME, USA) and were fed a standard chow with free access to water. Some mice were housed in the animal facility until they reached the age of twenty-six months old. Mouse primary hepatocytes were isolated by a two-step collagenase perfusion method as previously described and cultured overnight in Waymouth’s medium containing 100 units/mL penicillin, 100 g/mL streptomycin, 10% fetal bovine serum, 100 nM insulin, and 100 nM dexamethasone [11]. Cell viability was determined by trypan blue exclusion assay and was routinely greater than 85%. For the cell death assay, hepatocytes at a concentration of 1.4 × 10^5^ cells were plated onto 24-well microtiter plates (Falcon, Lincoln Park, NJ, USA). For immunoblot and subcellular fractionation analysis, hepatocytes were plated on 35 mm and 100 mm culture dishes at a concentration of 10^6^ and 3.5 × 10^6^ cells, respectively. For confocal microscopic studies, 2 × 10^5^ hepatocytes were cultured on 42 mm round glass coverslips in 60 mm culture dishes. All plates, dishes, and coverslips were coated with 0.1% Type 1 rat tail collagen. Hepatocytes were used after overnight culture in humidified 5% CO_2_ incubator at 37 °C. Experiments were performed in Krebs-Ringer- N-2 hydroxyethylpiperazine-N-2 ethanesulfonic acid buffer (KRH).

### 2.4. In Vitro Simulated I/R in Mouse Hepatocytes

Anoxia, nutrient depletion, and acidosis during ischemia were simulated by incubating hepatocytes in KRH at pH 6.2 in the anaerobic chamber (Coy Laboratory Products, Ann Arbor, MI, USA) [2,11]. After 2 h of ischemia, the anaerobic KRH at pH 6.2 was replaced with aerobic KRH at pH 7.4 to simulate reperfusion.

### 2.5. Adenoviral Infection

Some hepatocytes and livers were infected with an adenovirus expressing CAST-CFP (with a cytomegalovirus promoter) (Welgen inc, Worcester, MA, USA) in a hormonally defined medium as described previously [2,11]. An adenovirus harboring CFP (with a cytomegalovirus promoter) was used as a viral control. For the liver-specific delivery of CAST, some mice were infected with an adenoviral vector harboring CAST with α1-antitrypsin promoter (10 μL of 10^12^ viral particles/mL). 

### 2.6. Cell Death Assay

Hepatocyte death was assessed by PI fluorometry using a fluorescence plate reader (SpectraMax M2 or iD3; Molecular device, Sunnyvale, CA, USA), as described previously [2,11]. 

### 2.7. Autophagic Flux Measurement

To measure autophagic flux, hepatocytes were treated with 20 μM chloroquine (CQ), as described previously [20]. Changes in microtubule-associated protein light chain 3 (LC3) expression were monitored by immunoblot analysis in the presence or absence of CQ.

### 2.8. Immunoblot Analysis

Changes in protein expression were determined with immunoblot analysis as described previously [20]. Briefly, cell and tissue lysates were prepared in a radioimmunoprecipitation assay buffer with a protease/phosphatase inhibitor cocktail (Millipore Sigma). The protein concentration of lysates was determined with the bicinchoninic acid protein quantification kit (Thermo Fisher Scientific, Waltham, MA, USA). Protein samples of 8–22.5 μg were electrophoresed in 8–15% gradient polyacrylamide gels and transferred electrophoretically to a polyvinylidene fluoride membrane (Thermo Fisher Scientific, Waltham, MA, USA) or nitrocellulose membranes (GE Healthcare Life Sciences, Buckinghamshire, UK). Membranes were blocked in 5% nonfat milk and probed with primary antibodies including CAST (#4146, Cell Signaling Technology, Danvers, MA, USA), LC3-I/II (#2775, Cell Signaling Technology, Danvers, MA, USA), ATG12 (#4180, Cell Signaling Technology, Danvers, MA, USA), ATG5 (#A0856, Millipore Sigma), COX IV (#4844, Cell Signaling Technology, Danvers, MA, USA), VDAC (#4661, Cell Signaling Technology, Danvers, MA, USA), α-Tubulin (#sc-8035, Santa Cruz Biotechnology, Dallas, TX, USA), Lamin B1 (#9087, Cell Signaling Technology, Danvers, MA, USA), TNFα (#17590-1-AP, Proteintech, Rosemont, IL, USA), and β–actin (#A5441, Millipore Sigma). After incubating with appropriate horseradish peroxidase-conjugated secondary antibodies, specific protein bands were visualized using a Bio-Rad ChemiDoc MP trans-illuminator (Bio-Rad Life Sciences, Hercules, CA, USA). Protein expression was densitometrically quantified using IMAGE J software (NIH, Bethesda, MD, USA) and normalized to β–actin. 

### 2.9. In Vivo I/R

Hepatic ischemia in male C57BL/6 J mice was induced by occluding the portal triad for 30 min, as previously described [2]. Reperfusion was initiated by removing a microvascular clamp. Liver biopsies from the left lateral lobe were collected at 40 min of reperfusion and immediately frozen in liquid nitrogen.

### 2.10. Confocal and Multiphoton Microscopy

Confocal images of TMRM, calcein, and PI were collected using an inverted Zeiss 510 laser scanning confocal microscope [7]. Briefly, hepatocytes were incubated with 1 μM calcein/AM, 500 nM TMRM, and 30 μM PI to visualize MPT, mitochondrial membrane potential, and cell death, respectively. For intravital liver images, livers were labeled with Rd-123 (10 μM), as previously described [20]. After 40 min of reperfusion in vivo, the liver was placed over a glass coverslip on the stage of a Zeiss LSM510 equipped with a multiphoton microscope (Coherent Inc., Santa Clara, CA, USA). Images were collected with a 40 × water-immersion objective lens with an excitation of 780 nm. Fifteen to twenty images were randomly collected per liver. The relative fluorescence intensity of TMRM and Rd-123 was calculated using histogram analysis in Adobe Photoshop CS4 software (Adobe Systems, San Jose, CA, USA).

### 2.11. Statistical Analysis

Differences between means were compared by the Student’s *t*-test and the Mann–Whitney test using a level of significance of *p* < 0.05. Data were expressed as means ± standard error. Results were representative of at least three independent experiments. 

## 3. Results

### 3.1. Loss of CAST after I/R in Aged Livers

**Figure 1:**
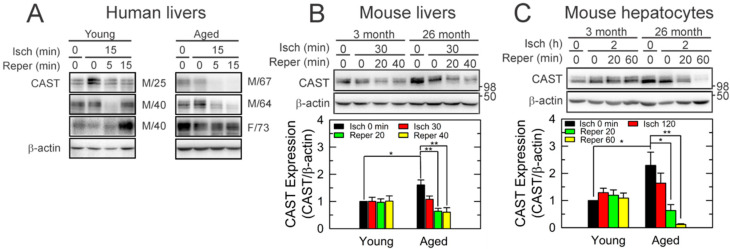
Loss of CAST after short I/R in aged livers. IB analysis of CAST in (**A**) human livers (*n* = 6), (**B**) mouse livers (*n* = 4), and (**C**) isolated mouse hepatocytes (*n* = 4). Liver tissues and hepatocytes at different ages were collected at the end of in vivo and in vitro ischemia (Isch) and during reperfusion (Reper). Changes in CAST were normalized to β-actin. * *p* < 0.05 and ** *p* < 0.01.

To examine whether aging alters CAST after I/R in human livers, non-tumorous specimens were collected from the patients who underwent liver resection. Six independent liver specimens from the male (M) and the female (F) patients at different ages were collected before and after 15 min of one episode of inflow occlusion, followed by 5 and 15 min after reperfusion (Figure 1A and Supplemental Table S1). Immunoblot (IB) analysis showed that CAST expression after 15 min of reperfusion was substantially lower than basal values (0 min of ischemia) in aged livers (64–73 years old), but not in young livers (25–40 years old). To test whether this age-dependent loss in CAST occurs in mice after I/R, we subjected livers from young (3-month-old) and aged (26-month-old) male C57 BL/6 J mice to 30 min of in vivo ischemia followed by 40 min of reperfusion (Figure 1B) [2,11]. Similar to human livers, in vivo I/R markedly decreased CAST only in aged livers. Next, hepatocytes were isolated from young and aged mice and exposed to 2 h of in vitro simulated ischemia, a mild ischemia causing minimal injury in young hepatocytes [20]. IB showed a progressive loss of CAST during I/R in aged cells (Figure 1C). After 60 min of reperfusion, CAST declined to 5% of basal levels, which did not occur to young cells. These data suggest that mild I/R depletes aged hepatocytes of CAST. 

### 3.2. Short-Lived CAST in Aged Hepatocytes

**Figure 2:**
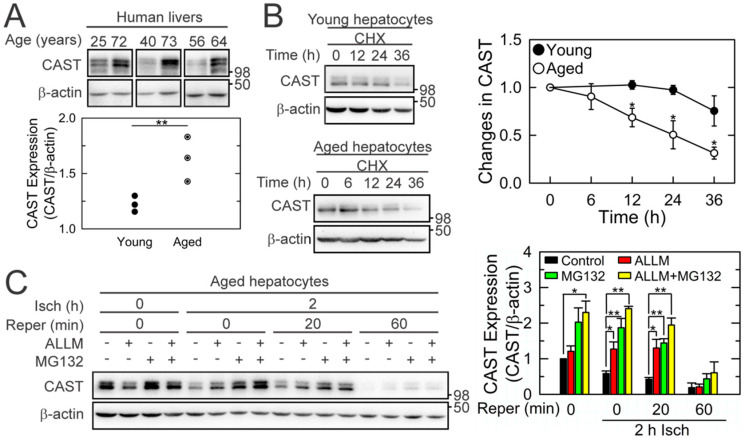
Decreased protein stability of CAST in aged livers. (**A**) CAST expression in human livers at different ages before the commencement of ischemia (*n* = 6). (**B**) Alterations of CAST expression in young and aged mouse hepatocytes in the presence of 35 μM CHX (*n* = 5). (**C**) Effects of 10 μM ALLM (calpain inhibitor) and 50 μM MG132 (proteasome inhibitor) on CAST expression in aged hepatocytes during I/R (*n* = 3). * *p* < 0.05 and ** *p* < 0.01.

Despite the age-dependent loss of CAST after I/R, unexpectedly, we observed a greater expression of basal CAST (before the commencement of ischemia) in the aged group than in the young group (Figure 1B,C and Figure 2A). To examine the stability of the CAST protein, hepatocytes at both ages were treated with cycloheximide (CHX), a translation inhibitor, under the normoxic condition and changes in CAST expression were analyzed by IB (Figure 2B). While a half-life time of CAST in young cells was estimated to be 84 h, approximately 50% of CAST disappeared within 25 h in the aged cells, suggesting that CAST in aged livers is intrinsically short-lived. The loss of CAST during the early phase of reperfusion in aged cells is likely associated with calpain activation and/or the ubiquitin/proteasome pathway because N-Acetyl-Leu-Leu-methional (ALLM), a calpain inhibitor [2], and/or MG132, substantially suppressed CAST loss during 20 min of reperfusion (Figure 2C). Neither agent, however, prevented CAST loss after 60 min of reperfusion. 

### 3.3. Cytoprotection by CAST Overexpression

**Figure 3:**
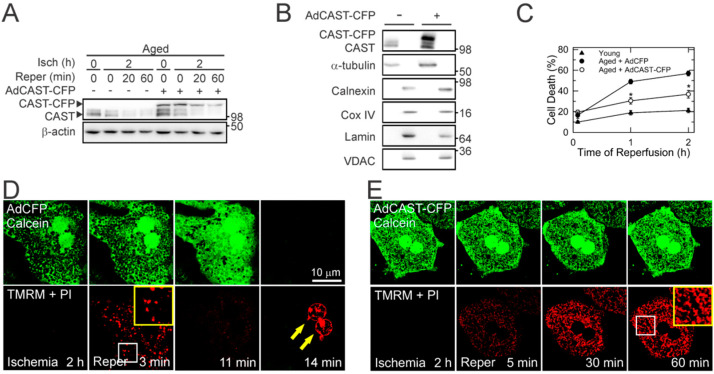
Cytoprotection by CAST overexpression. (**A**) IB analysis of CAST in aged hepatocytes during I/R with and without CAST overexpression. Representative IB from six independent cell isolations. (**B**) Subcellular fractionation of CAST with and without CAST overexpression. Calnexin, α-tubulin, cytochrome c oxidase (COX) IV and voltage-dependent anion channel (VDAC), and Lamin were used as a surrogate marker of endoplasmic reticulum, cytosol, mitochondria, and nucleus, respectively. (**C**) Cell death after I/R of young and aged hepatocytes in the presence of CAST overexpression (*n* = 9). Representative confocal images of calcein, TMRM, and PI in reperfused aged hepatocytes with (**E**) and without (**D**) CAST overexpression. Arrows indicate nuclear labeling of PI (necrotic cell death). The yellow squares are enlarged images of insets. * *p* < 0.05.

We next explored whether CAST depletion contributes to age-dependent cell death after reperfusion. We overexpressed CAST in aged hepatocytes with the adenovirus encoding cyan fluorescent protein (CFP)-tagged CAST (AdCAST-CFP). Adenovirus expressing CFP (AdCFP) was used for a viral control. CAST overexpression was confirmed by the appearance of the upper CAST band with slower mobility due to the presence of the CFP tag (Figure 3A). Subcellular fractionation assay indicated that both endogenous and exogenous CAST were localized predominantly in the cytosol (Figure 3B), consistent with previous reports [19,21]. Importantly, CAST overexpression significantly attenuated cell death in aged hepatocytes after I/R, implying a causative role of CAST loss in heightened I/R injury (Figure 3C). Similar to endogenous CAST, CAST-CFP progressively decreased after reperfusion, suggesting the presence of intrinsic cellular events causing CAST loss. 

### 3.4. Blockade of the MPT by CAST Overexpression

The onset of MPT is a cardinal event contributing to hepatocyte death after reperfusion [22,23]. We, therefore, sought to examine whether CAST overexpression can block the MPT. Laser scanning confocal microscopy was used to visualize the mitochondrial membrane potential (ΔΨm), MPT, and necrosis using TMRM, calcein, and PI, respectively (Figure 3D) [7,20]. Calcein is a green fluorescent dye with a molecular weight of 622.5 Da that is impermeable to polarized mitochondrial inner membranes. As a result, confocal images of calcein-loaded hepatocytes display a honeycomb appearance with dark round voids corresponding to individual polarized mitochondria that have not undergone the MPT. TMRM is a red fluorophore that electrophoretically accumulates in polarized mitochondria. PI labels nuclei of necrotic cells. After 2 h of ischemia, TMRM fluorescence was barely detectable due to anoxic depolarization in mitochondria while simultaneously excluding green fluorescent calcein, indicating a lack of the MPT during ischemia. Upon reperfusion, mitochondria transiently repolarized within 3 min. However, after 11 min, mitochondria began losing TMRM fluorescence and underwent the MPT, as evidenced by the redistribution of calcein from the cytosol to mitochondria (disappearance of dark voids). After 14 min, calcein fluorescence vanished and cell death ensued due to the rupture of plasma membranes, as shown by nuclear labeling of PI (arrows). In sharp contrast, MPT onset and cell death were blocked by CAST overexpression (Figure 3E), demonstrating that CAST overexpression prevents the MPT, mitochondrial depolarization, and necrosis in reperfused aged hepatocytes. Noticeably, a few mitochondria had elongated morphology with CAST overexpression, suggesting that CAST affects mitochondrial shape (yellow squares).

### 3.5. Restoration of Autophagy by CAST Overexpression

**Figure 4:**
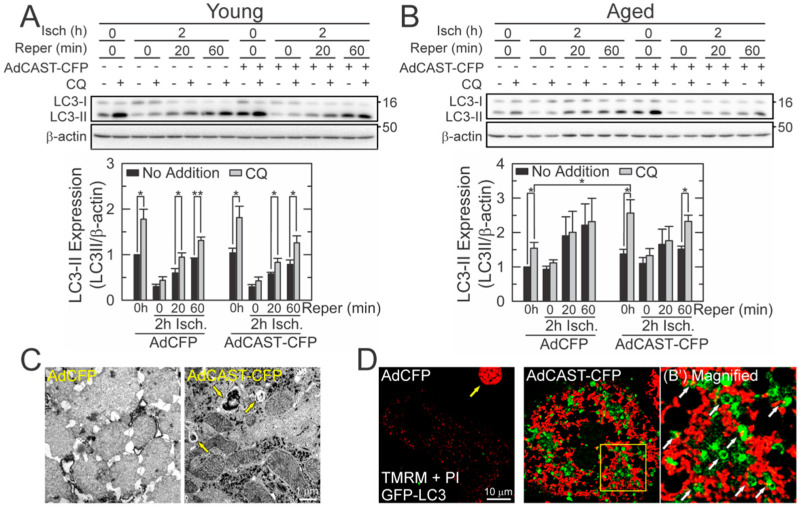
Restoration of autophagic flux in reperfused aged hepatocytes by CAST overexpression. Autophagic flux after 2 h of ischemia in young (**A**) and aged (**B**) hepatocytes with and without CAST overexpression (*n* = 5). Autophagic flux was measured by IB of LC3-II levels in the presence or absence of 20 µM CQ. Graph represents the quantification of LC3-II relative to its level at normoxia without CQ. (**C**) TEM images in reperfused aged hepatocytes with and without CAST overexpression. Yellow arrows indicate autophagic vehicles. (**D**) Representative confocal images of GFP-LC3 and TMRM after 2 h of ischemia followed by 30 min of reperfusion in aged hepatocytes. Yellow and white arrows indicate cell death and the onset of mitophagy, respectively. The right panel is an enlarged image of the square yellow inset in the middle panel. * *p* < 0.05 and ** *p* < 0.01.

Timely removal of aberrant mitochondria through autophagy is critical for cell survival. We have reported that reperfused aged hepatocytes have defective autophagy, leading to the development of MPT, mitochondrial failure, and, ultimately, necrosis [2,3]. Mitochondrial dysfunction after I/R can be mitigated by stimulating autophagy prior to the commencement of ischemia [2,20,24]. Accordingly, we investigated how CAST overexpression influences autophagy. Autophagic flux was determined biochemically using a lysosomal inhibitor, CQ (Figure 4A,B) [25,26]. The difference in the amount of LC3-II before and after CQ represents the net amount of autophagic cargo delivered to lysosomes [2]. Similar to previous results [2], aged cells lost autophagic flux after I/R whereas young cells sustained the flux. In aged cells, CAST overexpression not only restored autophagic flux after reperfusion but substantially enhanced the basal level of autophagy. On the contrary, CAST overexpression in young cells did not affect the flux before and during I/R. The importance of CAST in mitochondrial integrity and autophagy was also confirmed by transmission electron microscopy (TEM) (Figure 4C). After 30 min of reperfusion, CAST overexpression prevented structural distortion of mitochondria and promoted the formation of autophagic vesicles (yellow arrows). To directly visualize the onset of mitophagy, confocal images were collected after labeling aged cells with GFP-LC3, TMRM, and PI [2,11,20,24]. Control cells at 30 min after reperfusion displayed weak TMRM fluorescence and only a few autophagosomes (Figure 4D). The other cell had lost viability (yellow arrow). In clear contrast, the mitochondria with CAST overexpression remained polarized, many of which were surrounded by autophagosomes (white arrows), indicative of mitophagy onset [2,11]. Moreover, polarized mitochondria had both elongated and oval shapes. However, only the latter appeared to undergo mitophagy, implying the importance of mitochondrial size and shape in the onset of mitophagy. Together, our results firmly suggest that CAST is involved in sustaining autophagy, ΔΨm, and survivability in reperfused aged cells.

**Figure 5:**
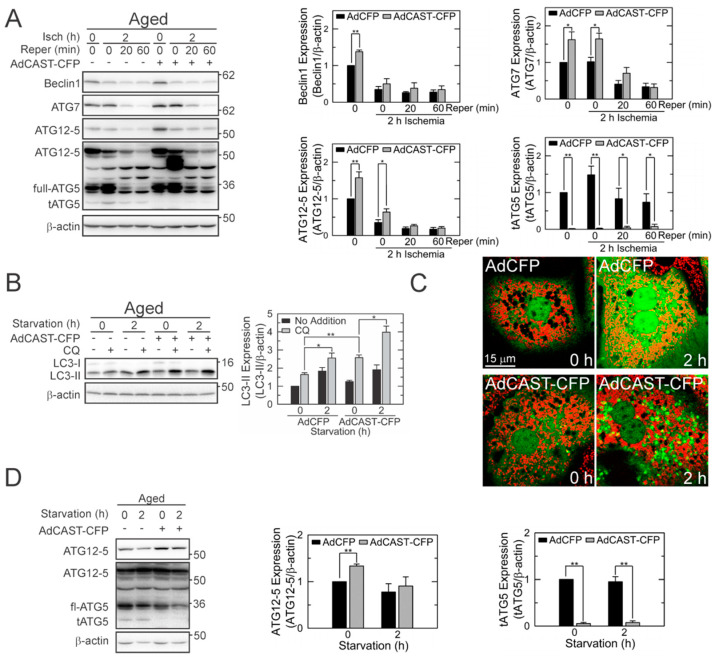
Suppression of tATG5 formation in reperfused aged hepatocytes by CAST overexpression. (**A**) IB analysis of ATGs during I/R of aged hepatocytes (*n* = 4). Graph represents the quantification of ATGs relative to their levels at normoxia without CAST overexpression. (**B**) Effects of CAST overexpression on autophagy induction under starvation conditions (*n* = 4). Changes in LC3-II were expressed relative to its level at 0 h without CAST overexpression. (**C**) Confocal images of GFP-LC3 and TMRM in aged hepatocytes after 2 h of starvation with (bottom panels) and without CAST overexpression (top panels). (**D**) IB analysis of ATGs in aged hepatocytes during 2 h of starvation (*n* = 3). Changes in individual ATGs were expressed relative to their levels at 0 h without CAST overexpression. * *p* < 0.05 and ** *p* < 0.01.

Calpains can hydrolyze autophagy proteins [2,17,18]. We, thus, investigated the effects of CAST overexpression on ATG expression. At normoxia, CAST overexpression significantly elevated the expression of Beclin-1, ATG7, and ATG12-5 (Figure 5A). Nevertheless, all three proteins substantially decreased after reperfusion. It has been reported that calpains cleave ATG5 into cytotoxic truncated form (tATG5) [17,18,27]. The generation of tATG5 was markedly subdued by CAST overexpression before and during I/R, connoting that CAST inhibits ATG5 cleavage. 

To further elucidate the mechanisms underlying the CAST-mediated enhancement of autophagy, we incubated aged hepatocytes in a nutrient-deficient buffer to simulate starvation, a condition that strongly induces autophagy (Figure 5B). CAST overexpression potentiated autophagy induction following 2 h of starvation. Parallel imaging experiments with GFP-LC3 and TMRM also confirmed the CAST-mediated potentiation of autophagy under nutrient-deficient conditions (Figure 5C). Of importance, CAST overexpression also markedly decreased tATG5 under this condition (Figure 5D). Collectively, these results support our conclusion that CAST overexpression prevents ATG5 cleavage and promotes autophagy in reperfused aged hepatocytes.

### 3.6. Mitochondrial Fusion by CAST Overexpression

**Figure 6:**
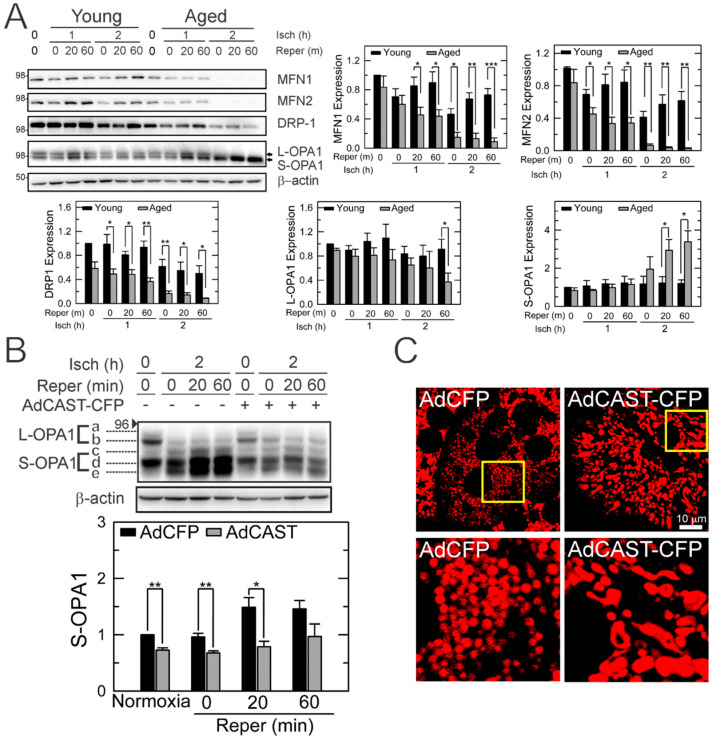
Altered MD in reperfused young and aged hepatocytes. (**A**) IB analysis of proteins related to MD after I/R in hepatocytes from different ages (*n* = 3). Changes in individual proteins were expressed relative to their levels in young hepatocytes at normoxia. (**B**) Determination of L- and S-OPA expression in reperfused aged hepatocytes before and after CAST overexpression (*n* = 3). Changes in S-OPA1 were expressed relative to its level at normoxia without CAST overexpression. (**C**) Confocal microscopy of TMRM-labelled mitochondria with and without CAST overexpression. Bottom panels represent enlarged images of the square inset in top panels. * *p* < 0.05, ** *p* < 0.01 and *** *p* < 0.001.

Mitochondria are highly dynamic organelles that continuously fuse and divide [28]. The balance between fusion and fission determines mitochondrial shape. In mammalian cells, fusion is governed by mitofusin (MFN) and optic atrophy 1 (OPA1) in mitochondrial outer and inner membranes, respectively, while fission is primarily mediated by dynamin-related protein 1 (DRP1) and fission-1-protein (FIS1) [28]. Like mitophagy, mitochondrial dynamics (MD) also plays a pivotal role in mitochondrial quality control. Accordingly, we analyzed MD proteins after I/R (Figure 6A). The basal expression of DRP1 was noticeably lower in aged hepatocytes than in young cells, although a statistical significance was not reached. Other MD proteins including MFN1, MFN2, and OPA1 were comparable between the two age groups. I/R significantly diminished MFN1 and 2 and DRP1 in aged cells. We observed that the short form of OPA1 (S-OPA1), a fission inducer [29,30,31,32], was increased in reperfused aged cells, whereas the long form of OPA1 (L-OPA1), a fusion inducer, was decreased after prolonged reperfusion, suggesting a shift of MD in reperfused aged cells. IB analysis of five unique OPA1 bands further revealed that I/R significantly enhanced the cleavage of L-OPA1 (band a and b) to S-OPA1 (band c and e), which was, however, suppressed by CAST overexpression. No other MD proteins were changed by AdCAST-CFP (data not shown). Altered mitochondrial shape by CAST was also confirmed by TMRM confocal microscopy (Figure 6C). Following CAST overexpression, aged cells had numerous interconnected mitochondria, supporting that CAST shifts the balance of MD toward fusion. Relative TMRM fluorescence intensity was similar before and after AdCAST-CFP treatment (47.5 ± 14. 8 vs. 60.43 ± 22.5), indicating minimal alterations in ΔΨm by CAST overexpression. Overall, these results suggest that CAST impacts both autophagy and mitochondrial fusion, two integral processes involved in mitochondrial quality control. 

### 3.7. Cytoprotection of Aged Livers by CAST Overexpression after In Vivo I/R

**Figure 7:**
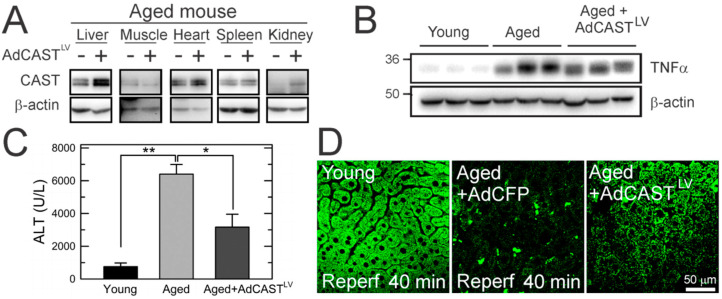
Cytoprotection of aged mouse livers by CAST overexpression after in vivo I/R. (**A**) Liver-specific delivery of adenoviral CAST. Change in (**B**) hepatic expression of tissue TNFα and (**C**) serum levels of ALT after 24 h of reperfusion in young and aged mouse livers with and without CAST overexpression (*n* = 3). (**D**) Multiphoton images of young and aged livers after I/R in vivo. * *p* < 0.05 and ** *p* < 0.01.

An adenoviral vector harboring CAST with an α1-antitrypsin promoter (AdCAST^LV^) facilitates the liver-specific delivery of CAST [33,34,35,36]. Some aged animals were intraperitoneally injected with this viral vector before the commencement of ischemia. IB analysis with the same animals showed that CAST was predominantly overexpressed in the liver with a minor increase in the kidney (Figure 7A). This viral vector did not affect the expression of CAST in skeletal muscles, hearts, and spleens. To extend our findings from hepatocytes to livers, young and aged mice were subjected to 70% partial hepatic ischemia by clamping the portal triad for 30 min. Compared to young mice, tissue TNFα (IB analysis) and serum ALT levels (colorimetric determination) after 24 h of reperfusion were significantly higher in aged mice, substantiating age-dependent I/R injury (Figure 7B,C). CAST overexpression markedly reduced serum ALT after I/R. Moreover, hepatic TNFα levels were decreased by AdCAST^LV^ treatment in two out of three aged mice, although a statistical significance was not reached. To test whether CAST overexpression suppresses mitochondrial depolarization, a salient feature of MPT [23], multiphoton images of rhodamine-123 (Rd-123), a mitochondrial potential indicator [2,37], were collected from young and aged mouse livers after 40 min of reperfusion (Figure 7D). The liver from a young mouse displayed numerous green punctae (polarized mitochondria) in hepatocytes. The aged liver with a control virus, however, exhibited diffuse and weak Rd-123 fluorescence, signifying a mitochondrial depolarization and bioenergetic failure [2,38]. The relative fluorescence intensity of Rd-123 was significantly decreased from 41.5 ± 12.1 (young control) to 0.9 ± 1.1 (aged control) after reperfusion. In sharp contrast, CAST overexpression attenuated reperfusion-induced loss in ΔΨm. Compared to the aged control, the relative fluorescence intensity of Rd-123 was markedly greater in the AdCAST^LV^ group (0.9 ± 1.1 vs. 28.9 ± 6.3). Altogether, these results support our conclusion that CAST overexpression is cytoprotective against mitochondrial dysfunction in reperfused aged livers.

## 4. Discussion

I/R injury is unavoidable during liver surgery and remains a serious threat to elderly patients because aged livers have a significantly lower reparative capacity following reperfusion. In the present study, we investigated the roles of CAST in age-dependent I/R injury using young and aged mouse and human livers. Unexpectedly, we found a higher basal expression of CAST in aged livers than in young livers. However, when exposed to short I/R, modest stress inducing minimal injury to young livers, aged livers rapidly lost CAST, leading to the stimulation of calpains, defective autophagy, accumulation of dysfunctional mitochondria, and, ultimately, hepatocyte death. The importance of CAST in the survivability of reperfused aged hepatocytes is supported by our findings; (1) short and modest I/R depletes aged livers and hepatocytes of CAST, which does not occur in young counterparts (Figure 1). (2) Despite its higher basal level, CAST in aged livers is intrinsically short-lived and rapidly declines after I/R (Figure 2). (3) CAST overexpression suppresses defective autophagy, MPT onset, mitochondrial depolarization, and cell death after reperfusion (Figure 3, Figure 4 and Figure 5). (4) As well as autophagy induction, CAST overexpression shifts MD toward mitochondrial fusion (Figure 6). (5) CAST overexpression protects aged livers from in vivo I/R injury (Figure 7). Collectively, our results suggest that CAST is indispensable for the survival of aged livers after I/R. 

Uncontrolled stimulation of calpains incites irreversible tissue injury through the degradation of key structural and functional proteins. We have reported that Ca^2+^ overloading during I/R over-activates calpains, resulting in defective autophagy and hepatocyte death [2,11,20,24,39]. Our results here substantiated a causative role of calpains in I/R injury. Under normal conditions, the over-activation of calpains rarely occurs due to the presence of CAST. The fact that one CAST blocks four calpains through its binding to two penta-EF domains and one catalytic cleft of calpains [40,41] raised the question as to how reperfused hepatocytes could develop calpain-mediated cellular damage even in the presence of such a powerful endogenous calpain inhibitor. We observed that despite its higher basal level, CAST in aged livers was short-lived. 

CAST depletion during I/R in aged hepatocytes appears to be, at least in part, due to the activation of calpains and/or proteasomes since calpain and a proteasome inhibitor recovered CAST during the early stage of reperfusion. While the basal activity of calpains, as assessed by the formation of auto-hydrolyzed fragments (18- to 54-kDa) [24], was comparable between young and aged cells, a substantial increase in calpain activity was detected only after reperfusion in aged cells (data not shown). Indeed, aging per se minimally impacts the viability and functionality of hepatocytes, but a noticeable cellular injury in aged cells occurs after exposure to stresses [2]. Of note, aged cells lost CAST after prolonged reperfusion even with calpain and proteasome inhibitors, indicating the presence of other mechanisms behind CAST depletion after reperfusion. One possible mechanism could be a distinct status of acetylation/deacetylation of CAST in aged cells. Compared to young hepatocytes, Sirtuin 1 (SIRT1) expression is significantly lower in aged cells [39]. Bioinformatic analysis predicts that CAST has 16 acetyl-Lys (K) sites (from K42 to K739) that can be deacetylated by SIRT1 [42]. In the pilot studies with immunoprecipitation experiments, we observed that the ablation of SIRT1 markedly increased levels of acetylated CAST. A CHX study also revealed that a half-life time of CAST in SIRT1-null hepatocytes was much shorter than that in wild-type cells (data not shown), implying that the reduced stability of CAST in aged cells could result from abundant hyperacetylated CAST. The importance of acetylation status in protein stability has been reported [43,44]. Thus, higher levels of basal CAST in aged cells may represent a compensatory mechanism that counteracts intrinsically unstable CAST in aged livers. 

Functional autophagy is critical for cell survival during I/R [2,11,39]. We showed that CAST overexpression in aged hepatocytes restored autophagic flux and suppressed cell death after I/R, confirming an integral role of autophagy in hepatocyte viability after I/R. CAST overexpression significantly increased the expression of Beclin-1, ATG7, and ATG12-5 before reperfusion. However, their expressions substantially declined after reperfusion. It is unknown how reperfused aged cells with lower levels of key autophagy proteins could efficaciously drive autophagy. Intriguingly, CAST overexpression significantly reduced tATG5 during I/R. Calpains cleave full-length ATG5 (~33 kDa) at Thr193 into tATG5 (~24 kDa) that subsequently translocates to mitochondria, leading to a loss in ΔΨm [17,27]. As a further decline in ΔΨm worsens a cellular ATP shortage in reperfused aged hepatocytes, CAST-mediated suppression of tATG5 formation is likely to prevent additional loss in ΔΨm, thereby allowing cells to reserve ATP that could be used for the execution of energy-dependent cellular processes such as autophagy. In line with this notion, we observed that CAST overexpression prevented tATG5 formation and promoted autophagy under starvation conditions. 

Another interesting finding is that CAST overexpression shifts MD toward fusion. While cytoprotective, CAST overexpression did not recover MFN1, MFN2, and DRP1 after I/R. Among MD proteins, only S-OPA1 expression was profoundly diminished by CAST overexpression. L-OPA1 induces fusion, whereas S-OPA1 triggers fission [45]. Excessive conversion of L-OPA1 into S-OPA1 is known to induce tissue injury [46,47,48]. Under non-lethal conditions, healthy mitochondria fuse or interconnect with suboptimal mitochondria to sustain bioenergetic efficiency [12]. This adaptive morphological change rescues 80% of the mitochondrial DNA abnormalities and increases mitochondrial oxidative phosphorylation through sharing individual mitochondrial contents [49,50]. Indeed, strategies promoting mitochondrial fusion exert cytoprotection against nutrient insufficiency [51], oxidative stress [52], neurodegeneration [53], cardiac [54] and renal reperfusion injury [55]. Mitochondrial fusion has been proposed to serve as the first line of defense against acute stress [14]. Suboptimal mitochondrion that fails to fuse with healthy mitochondrion is eliminated by mitophagy later [14]. As autophagy is a highly energy-consuming process [56,57], elevated demand for autophagy becomes an additional burden to energetically compromised cells. If damaged mitochondria continue to escape the fusion process, the autophagic burden exceeds the cellular capacity of autophagy. Dysfunctional mitochondria accumulate and cell death ensues thereafter. Hence, mitochondrial fusion triggered by CAST appears to be another protective mechanism by which the autophagic and energetic burden is relieved in hepatocytes.

Numerous synthetic calpain inhibitors have been developed, yet their clinical use is limited due to a lack of specificity and side effects [58]. The present study suggests that CAST may be a potential therapeutic target to reduce I/R injury. Despite the development of a few CAST-based inhibitors such as subdomain B peptide and oligoarginine-conjugated peptides, their clinical applicability is far from ideal mainly because these peptidomimetic compounds fail to generate α-helical and β-barrel structures upon their binding to calpains, key structural motives required for calpain-specific inhibition [59]. Thus, short peptide sequences in CAST subdomains may not fully mimic the efficacy of CAST, and gene therapy delivering a full sequence of the CAST gene could be a therapeutic strategy to decrease I/R injury in aged livers.

In summary, CAST in aged hepatocytes is abundant yet short-lived and becomes rapidly depleted after I/R. CAST loss after I/R leads to the stimulation of calpains, accumulation of tATG5, impaired autophagy, MPT onset, and hepatocyte death, all of which are suppressed by CAST overexpression. Moreover, CAST shifts mitochondrial morphology toward an elongated shape in order to sustain hepatocyte viability after I/R. These findings suggest that CAST may be a therapeutic target to ameliorate I/R injury in elderly patients undergoing liver surgery.

## Data Availability

The data presented in this study are available on request to J.-S.K.

## Supplementary Materials

The following are available online at https://www.mdpi.com/article/10.3390/cells10081863/s1, Table S1: Patients’ Characteristics.

## References

[1] Veering B.T. (1999). Management of anaesthesia in elderly patients. Curr. Opin. Anaesthesiol..

[2] Wang J.H., Ahn I.S., Fischer T.D., Byeon J.I., Dunn W.A., Behrns K.E., Leeuwenburgh C., Kim J.-S. (2011). Autophagy suppresses age-dependent ischemia and reperfusion injury in livers of mice. Gastroenterology.

[3] Wang J.H., Behrns K.E., Leeuwenburgh C., Kim J.-S. (2012). Critical role of autophagy in ischemia/reperfusion injury to aged livers. Autophagy.

[4] Czaja M.J., Ding W.X., Donohue T.M., Friedman S.L., Kim J.-S., Komatsu M., Lemasters J.J., Lemoine A., Lin J.D., Ou J.H. (2013). Functions of autophagy in normal and diseased liver. Autophagy.

[5] Lee S., Kim J.-S. (2014). Mitophagy: Therapeutic potentials for liver disease and beyond. Toxicol. Res..

[6] Go K.L., Lee S., Zendejas I., Behrns K.E., Kim J.-S. (2015). Mitochondrial Dysfunction and Autophagy in Hepatic Ischemia/Reperfusion Injury. Biomed. Res. Int..

[7] Qian T., Nieminen A.L., Herman B., Lemasters J.J. (1997). Mitochondrial permeability transition in pH-dependent reperfusion injury to rat hepatocytes. Am. J. Physiol..

[8] Nieminen A.L., Saylor A.K., Tesfai S.A., Herman B., Lemasters J.J. (1995). Contribution of the mitochondrial permeability transition to lethal injury after exposure of hepatocytes to t-butylhydroperoxide. Biochem. J..

[9] Kon K., Kim J.-S., Jaeschke H., Lemasters J.J. (2004). Mitochondrial permeability transition in acetaminophen-induced necrosis and apoptosis of cultured mouse hepatocytes. Hepatology.

[10] McGill M.R., Sharpe M.R., Williams C.D., Taha M., Curry S.C., Jaeschke H. (2012). The mechanism underlying acetaminophen-induced hepatotoxicity in humans and mice involves mitochondrial damage and nuclear DNA fragmentation. J. Clin. Investig..

[11] Kim J.-S., Nitta T., Mohuczy D., O’Malley K.A., Moldawer L.L., Dunn W.A., Behrns K.E. (2008). Impaired autophagy: A mechanism of mitochondrial dysfunction in anoxic rat hepatocytes. Hepatology.

[12] Gomes L.C., Di Benedetto G., Scorrano L. (2011). During autophagy mitochondria elongate, are spared from degradation and sustain cell viability. Nat. Cell Biol..

[13] Youle R.J., van der Bliek A.M. (2012). Mitochondrial fission, fusion, and stress. Science.

[14] Twig G., Elorza A., Molina A.J., Mohamed H., Wikstrom J.D., Walzer G., Stiles L., Haigh S.E., Katz S., Las G. (2008). Fission and selective fusion govern mitochondrial segregation and elimination by autophagy. EMBO J..

[15] Byrne A.M., Lemasters J.J., Nieminen A.L. (1999). Contribution of increased mitochondrial free Ca^2+^ to the mitochondrial permeability transition induced by tert-butylhydroperoxide in rat hepatocytes. Hepatology.

[16] Kim J.-S., Wang J.H., Lemasters J.J. (2012). Mitochondrial permeability transition in rat hepatocytes after anoxia/reoxygenation: Role of Ca2+-dependent mitochondrial formation of reactive oxygen species. Am. J. Physiol. Gastrointest. Liver Physiol..

[17] Yousefi S., Perozzo R., Schmid I., Ziemiecki A., Schaffner T., Scapozza L., Brunner T., Simon H.U. (2006). Calpain-mediated cleavage of Atg5 switches autophagy to apoptosis. Nat. Cell Biol..

[18] Norman J.M., Cohen G.M., Bampton E.T. (2010). The in vitro cleavage of the hAtg proteins by cell death proteases. Autophagy.

[19] Wendt A., Thompson V.F., Goll D.E. (2004). Interaction of calpastatin with calpain: A review. Biol. Chem..

[20] Biel T.G., Lee S., Flores-Toro J.A., Dean J.W., Go K.L., Lee M.H., Law B.K., Law M.E., Dunn W.A., Zendejas I. (2016). Sirtuin 1 suppresses mitochondrial dysfunction of ischemic mouse livers in a mitofusin 2-dependent manner. Cell Death Differ..

[21] Limaye P.B., Bhave V.S., Palkar P.S., Apte U.M., Sawant S.P., Yu S., Latendresse J.R., Reddy J.K., Mehendale H.M. (2006). Upregulation of calpastatin in regenerating and developing rat liver: Role in resistance against hepatotoxicity. Hepatology.

[22] Kim J.-S., He L., Qian T., Lemasters J.J. (2003). Role of the mitochondrial permeability transition in apoptotic and necrotic death after ischemia/reperfusion injury to hepatocytes. Curr. Mol. Med..

[23] Kim J.-S., He L., Lemasters J.J. (2003). Mitochondrial permeability transition: A common pathway to necrosis and apoptosis. Biochem. Biophys. Res. Commun..

[24] Kim J.-S., Wang J.H., Biel T.G., Kim D.S., Flores-Toro J.A., Vijayvargiya R., Zendejas I., Behrns K.E. (2013). Carbamazepine suppresses calpain-mediated autophagy impairment after ischemia/reperfusion in mouse livers. Toxicol. Appl. Pharmacol..

[25] Mizushima N., Klionsky D.J. (2007). Protein turnover via autophagy: Implications for metabolism. Annu. Rev. Nutr..

[26] Mizushima N., Yoshimori T., Levine B. (2010). Methods in mammalian autophagy research. Cell.

[27] Xia H.G., Zhang L., Chen G., Zhang T., Liu J., Jin M., Ma X., Ma D., Yuan J. (2010). Control of basal autophagy by calpain1 mediated cleavage of ATG5. Autophagy.

[28] Liesa M., Shirihai O.S. (2013). Mitochondrial dynamics in the regulation of nutrient utilization and energy expenditure. Cell Metab..

[29] Ehses S., Raschke I., Mancuso G., Bernacchia A., Geimer S., Tondera D., Martinou J.C., Westermann B., Rugarli E.I., Langer T. (2009). Regulation of OPA1 processing and mitochondrial fusion by m-AAA protease isoenzymes and OMA1. J. Cell Biol..

[30] Ishihara N., Fujita Y., Oka T., Mihara K. (2006). Regulation of mitochondrial morphology through proteolytic cleavage of OPA1. EMBO J..

[31] Song Z., Chen H., Fiket M., Alexander C., Chan D.C. (2007). OPA1 processing controls mitochondrial fusion and is regulated by mRNA splicing, membrane potential, and Yme1L. J. Cell Biol..

[32] Varanita T., Soriano M.E., Romanello V., Zaglia T., Quintana-Cabrera R., Semenzato M., Menabo R., Costa V., Civiletto G., Pesce P. (2015). The OPA1-dependent mitochondrial cristae remodeling pathway controls atrophic, apoptotic, and ischemic tissue damage. Cell Metab..

[33] Hafenrichter D.G., Ponder K.P., Rettinger S.D., Kennedy S.C., Wu X., Saylors R.S., Flye M.W. (1994). Liver-directed gene therapy: Evaluation of liver specific promoter elements. J. Surg. Res..

[34] Kramer M.G., Barajas M., Razquin N., Berraondo P., Rodrigo M., Wu C., Qian C., Fortes P., Prieto J. (2003). In vitro and in vivo comparative study of chimeric liver-specific promoters. Mol. Ther..

[35] Hafenrichter D.G., Wu X., Rettinger S.D., Kennedy S.C., Flye M.W., Ponder K.P. (1994). Quantitative evaluation of liver-specific promoters from retroviral vectors after in vivo transduction of hepatocytes. Blood.

[36] Kelsey G.D., Povey S., Bygrave A.E., Lovell-Badge R.H. (1987). Species- and tissue-specific expression of human alpha 1-antitrypsin in transgenic mice. Genes Dev..

[37] Zhong Z., Theruvath T.P., Currin R.T., Waldmeier P.C., Lemasters J.J. (2007). NIM811, a mitochondrial permeability transition inhibitor, prevents mitochondrial depolarization in small-for-size rat liver grafts. Am. J. Transplant..

[38] Imberti R., Nieminen A.L., Herman B., Lemasters J.J. (1993). Mitochondrial and glycolytic dysfunction in lethal injury to hepatocytes by t-butylhydroperoxide: Protection by fructose, cyclosporin A and trifluoperazine. J. Pharmacol. Exp. Ther..

[39] Chun S.K., Lee S., Flores-Toro J., U R.Y., Yang M.J., Go K.L., Biel T.G., Miney C.E., Pierre Louis S., Law B.K. (2018). Loss of sirtuin 1 and mitofusin 2 contributes to enhanced ischemia/reperfusion injury in aged livers. Aging Cell.

[40] Hanna R.A., Campbell R.L., Davies P.L. (2008). Calcium-bound structure of calpain and its mechanism of inhibition by calpastatin. Nature.

[41] Moldoveanu T., Gehring K., Green D.R. (2008). Concerted multi-pronged attack by calpastatin to occlude the catalytic cleft of heterodimeric calpains. Nature.

[42] Zhai Z., Tang M., Yang Y., Lu M., Zhu W.G., Li T. (2017). Identifying Human SIRT1 Substrates by Integrating Heterogeneous Information from Various Sources. Sci. Rep..

[43] Jeong J.W., Bae M.K., Ahn M.Y., Kim S.H., Sohn T.K., Bae M.H., Yoo M.A., Song E.J., Lee K.J., Kim K.W. (2002). Regulation and destabilization of HIF-1alpha by ARD1-mediated acetylation. Cell.

[44] Zhou Q., Agoston A.T., Atadja P., Nelson W.G., Davidson N.E. (2008). Inhibition of histone deacetylases promotes ubiquitin-dependent proteasomal degradation of DNA methyltransferase 1 in human breast cancer cells. Mol. Cancer Res..

[45] Wai T., Garcia-Prieto J., Baker M.J., Merkwirth C., Benit P., Rustin P., Ruperez F.J., Barbas C., Ibanez B., Langer T. (2015). Imbalanced OPA1 processing and mitochondrial fragmentation cause heart failure in mice. Science.

[46] MacVicar T., Langer T. (2016). OPA1 processing in cell death and disease—The long and short of it. J. Cell Sci..

[47] Sun Y., Xue W., Song Z., Huang K., Zheng L. (2016). Restoration of Opa1-long isoform inhibits retinal injury-induced neurodegeneration. J. Mol. Med..

[48] Baburamani A.A., Hurling C., Stolp H., Sobotka K., Gressens P., Hagberg H., Thornton C. (2015). Mitochondrial Optic Atrophy (OPA) 1 Processing Is Altered in Response to Neonatal Hypoxic-Ischemic Brain Injury. Int. J. Mol. Sci..

[49] Yoneda M., Miyatake T., Attardi G. (1994). Complementation of mutant and wild-type human mitochondrial DNAs coexisting since the mutation event and lack of complementation of DNAs introduced separately into a cell within distinct organelles. Mol. Cell Biol..

[50] Nakada K., Inoue K., Ono T., Isobe K., Ogura A., Goto Y.I., Nonaka I., Hayashi J.I. (2001). Inter-mitochondrial complementation: Mitochondria-specific system preventing mice from expression of disease phenotypes by mutant mtDNA. Nat. Med..

[51] Rambold A.S., Kostelecky B., Elia N., Lippincott-Schwartz J. (2011). Tubular network formation protects mitochondria from autophagosomal degradation during nutrient starvation. Proc. Natl. Acad. Sci. USA.

[52] Chou C.H., Lin C.C., Yang M.C., Wei C.C., Liao H.D., Lin R.C., Tu W.Y., Kao T.C., Hsu C.M., Cheng J.T. (2012). GSK3beta-mediated Drp1 phosphorylation induced elongated mitochondrial morphology against oxidative stress. PLoS ONE.

[53] Chen H., McCaffery J.M., Chan D.C. (2007). Mitochondrial fusion protects against neurodegeneration in the cerebellum. Cell.

[54] Ong S.B., Subrayan S., Lim S.Y., Yellon D.M., Davidson S.M., Hausenloy D.J. (2010). Inhibiting mitochondrial fission protects the heart against ischemia/reperfusion injury. Circulation.

[55] Brooks C., Wei Q., Cho S.G., Dong Z. (2009). Regulation of mitochondrial dynamics in acute kidney injury in cell culture and rodent models. J. Clin. Investig..

[56] Schellens J.P., Vreeling-Sindelarova H., Plomp P.J., Meijer A.J. (1988). Hepatic autophagy and intracellular ATP. A morphometric study. Exp. Cell Res..

[57] Plomp P.J., Wolvetang E.J., Groen A.K., Meijer A.J., Gordon P.B., Seglen P.O. (1987). Energy dependence of autophagic protein degradation in isolated rat hepatocytes. Eur. J. Biochem..

[58] Ono Y., Saido T.C., Sorimachi H. (2016). Calpain research for drug discovery: Challenges and potential. Nat. Rev. Drug Discov..

[59] Dókus L.E., Yousef M., Bánóczi Z. (2020). Modulators of calpain activity: Inhibitors and activators as potential drugs. Expert Opin. Drug Discov..

