# Supplementary material for: Critical Roles of Calpastatin in Ischemia/Reperfusion Injury in Aged Livers
Source: Cells. 2021 Jul 23;10(8):1863. doi: 10.3390/cells10081863 (PMC8394464; doi:10.3390/cells10081863)
Supplement: Supplementary file 1 [file cells-10-01863-s001.zip › cells-1283008-supplementary.pdf]

**Supplemental Table. Patients' Characteristics**

| **Patient** | 1 | 2 | 3 | 4 | 5 | 6 | 7 | 8 | 9 | 10 | 11 | 12 |
|---|---|---|---|---|---|---|---|---|---|---|---|---|
| **Age** | 25 | 40 | 40 | 67 | 64 | 73 | 25 | 72 | 40 | 73 | 56 | 64 |
| **Sex** | M | M | M | M | M | F | F | F | M | M | M | M |
| **Cause of resection** | HCC | HCC | HCC | HCC | HCC | HCC | HCC | HCC | HCC | HCC | HCC | HCC |
| **Previous condition** | HCC | CC | GD | None | HCV | HC | None | HC | None | None | CC | None |
| **I/R** | + | + | + | + | + | + | - | - | - | - | - | - |

Abbreviations: HCC, Hepatocellular cancer; CC, Colorectal cancer; GD, Glycoprotein disorder; HCV, Hepatitis C; HC, Hepatic cysts; I/R, ischemia/reperfusion through the Pringle maneuver
